# Improved Electrochemical Performance Based on Nanostructured SnS_2_@CoS_2_–rGO Composite Anode for Sodium-Ion Batteries

**DOI:** 10.1007/s40820-018-0200-x

**Published:** 2018-04-13

**Authors:** Xia Wang, Xueying Li, Qiang Li, Hongsen Li, Jie Xu, Hong Wang, Guoxia Zhao, Lisha Lu, Xiaoyu Lin, Hongliang Li, Shandong Li

**Affiliations:** 1 College of Physics, Key Laboratory of Photonics Materials and Technology in Universities of Shandong, and Laboratory of Fiber Materials and Modern Textile, the Growing Base for State Key Laboratory, Qingdao University, Qingdao, 266071 People’s Republic of China; 2 Institute of Materials for Energy and Environment, Qingdao University, Qingdao, 266071 People’s Republic of China

**Keywords:** SnS_2_ nanosheets, CoS_2_ nanoparticles, Reduced graphene oxide (rGO), Sodium-ion batteries (SIBs)

## Abstract

A promising anode material composed of SnS_2_@CoS_2_ flower-like spheres assembled from SnS_2_ nanosheets and CoS_2_ nanoparticles accompanied by reduced graphene oxide (rGO) was fabricated by a facile hydrothermal pathway. The presence of rGO and the combined merits of SnS_2_ and CoS_2_ endow the SnS_2_@CoS_2_–rGO composite with high conductivity pathways and channels for electrons and with excellent properties as an anode material for sodium-ion batteries (SIBs). A high capacity of 514.0 mAh g^−1^ at a current density of 200 mA g^−1^ after 100 cycles and a good rate capability can be delivered. The defined structure and good sodium-storage performance of the SnS_2_@CoS_2_–rGO composite demonstrate its promising application in high-performance SIBs.

**Electronic supplementary material:**

The online version of this article (10.1007/s40820-018-0200-x) contains supplementary material, which is available to authorized users.

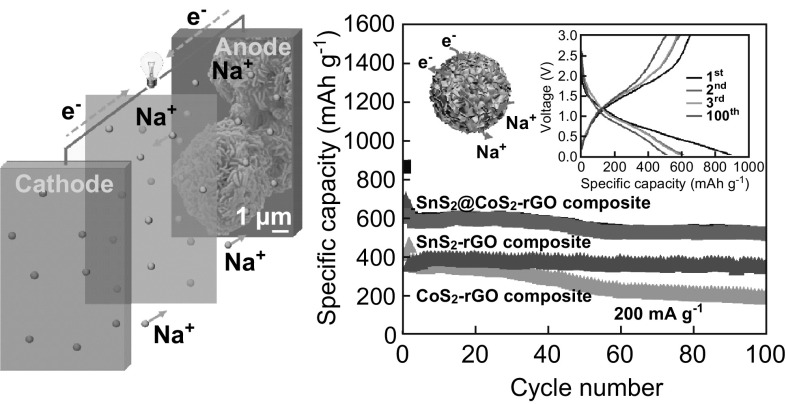

## Highlights

- A flower-like nanostructured composition of SnS_2_@CoS_2_ spheres associated with reduced graphene oxide (rGO) was prepared by a facile method.
- The anode based on SnS_2_@CoS_2_–rGO composite exhibited excellent cycling stability and rate capability for sodium-ion batteries (SIBs).

## Introduction

As promising alternatives to lithium-ion batteries (LIBs), sodium-ion batteries (SIBs) have attracted increasing attention, by virtue of the low cost and natural abundance of sodium salts, in combination with a suitable redox potential (− 2.71 V vs. the standard hydrogen electrode) [1–4]. Nonetheless, the larger radius and slower reaction kinetics of sodium ions compared to those of lithium ions would cause large volume variation and large polarization of anode materials, which lead to poor cycling performance and low reversible capacity [5–8]. Therefore, it is still challenging to find suitable anode materials with excellent sodium-storage performance.

Previously, a large number of anode materials, including metals [9], metal oxides [10], metal sulfides [11, 12], and metal selenides [13] have been investigated as potential anode materials with high capacity for SIBs. In particular, metal sulfides such as MoS_2_ [14], CoS_2_ [15], Fe_1−*x*_S [16], Co_3_S_4_ [17], In_2_S_3_ [18], SnS [19, 20], and Sb_2_S_3_ [21, 22] have attracted attention for SIBs owing to their high theoretical capacity, better reversibility and higher electrical conductivity than those of metal oxides. Among them, SnS_2_ with two-dimensional (2D) layered crystal structure is regarded as a potential anode material for SIBs, based on its large interlayer spacing along the *c*-axis, which offers an effective diffusion path for the intercalation and exfoliation of Na^+^ in the charge/discharge process; hence, it could possess fast ion diffusion kinetics and high specific capacity [23, 24]. Nevertheless, owing to low intrinsic electrical conductivity (8.21 × 10^−4^ S cm^−1^) as well as large volume variation during cycling, SnS_2_ is demonstrated poor cycling stability and rate capability. Various measures have been employed to alleviate this issue, such as reduction of crystal size [25], fabrication of various nanoarchitectures [26], and formation of composites with conductive materials [27, 28].

CoS_2_ possesses good thermal stability and low cost, and hence it is considered a suitable anode material for SIBs [29]. However, it exhibits inferior electrochemical properties, which may arise from its sluggish ion transport kinetics with low electrical conductivity (1.63 × 10^−5^ S cm^−1^) [30]. In view of this, combining SnS_2_ and CoS_2_ into rationally designed composite architectures could possibly provide synergistic merits. Furthermore, in the discharge/charge process, the Co generated in the conversion reaction of CoS_2_ can enhance the reversibility of SnS_2_ [31]. A hierarchical composite assembled from different metal sulfides could not only supply rich redox chemistry and synergistically display the merits of each component, but could also benefit from the interactions of the individual components [30, 32]. For instance, Dong et al. successfully fabricated a polyhedral composite employing a zeolitic imidazolate framework (ZIF-8) as precursor with a ZnS inner-core and Sb_2_S_3_/C double-shell as an anode material for SIBs [11]. Compared to ZnS@C, the polyhedron composite presented significantly improved sodium-storage performance with good cycle stability and high specific capacity, ascribed to the cooperative contributions of ZnS, Sb_2_S_3_, and C components and a stable structure with sufficient space to alleviate volume variation on cycling. Geng et al. synthesized cobalt sulfide/molybdenum disulfide (Co_9_S_8_/MoS_2_) yolk–shell spheres, which possessed outstanding lithium/sodium-storage performance due to coordination of the yolk–shell structure and the well-distributed mixture consisting of Co_9_S_8_ and MoS_2_ nanocrystals [30]. Fe_3_O_4_/Fe_1−*x*_S@C@MoS_2_ nanosheets composed of Fe_3_O_4_/Fe_1−*x*_S nanoparticles inserted in carbon nanosheets and encapsulated by MoS_2_ were fabricated by Pan et al., and presented excellent electrochemical performance owing to the synergistic effects of each component [33]. Moreover, Lin et al. reported a novel bicontinuous carbon wrapped NiS_2_@CoS_2_ hetero-nanocrystal hierarchical structure, which exhibited superior rate capability and cycle stability for SIBs, benefiting from interconnected porous structures and structural integrity due to double carbon frameworks [34]. Therefore, in order to further develop a metal sulfide composite with better electrochemical performance, their incorporation into a carbon matrix such as carbon nanotubes, graphene, or reduced graphene oxide (rGO) has been proposed, because rGO and graphene exhibit superior electroconductivity, pronounced chemical stability, remarkable flexibility, and high surface area to buffer the mechanical stress experienced during the conversion process [35]. Therefore, the growth of SnS_2_ and CoS_2_ on rGO to form a SnS_2_@CoS_2_–rGO composite should demonstrate good sodium-storage performance. To our knowledge, this SnS_2_@CoS_2_–rGO composite has not been previously employed as an anode material for SIBs.

In the present study, a SnS_2_@CoS_2_–rGO composite was synthesized successfully by a hydrothermal procedure, in which SnS_2_@CoS_2_ flower-like spheres assembled by SnS_2_ nanosheets and CoS_2_ nanoparticles were composited with rGO sheets. The sodium-storage performance of the composite was evaluated when used as an anode material. An enhanced reversible capacity, long cycling stability, and good rate capability of the composite were obtained. Therefore, the SnS_2_@CoS_2_–rGO composite has good potential for use as a highly performing anode material in SIBs.

## Experimental Section

### Synthetic Procedures of the SnS_2_@CoS_2_–rGO Composite

Firstly, graphene oxide (GO) was fabricated through a modified Hummers method [36]. The SnS_2_@CoS_2_–rGO composite was prepared by a hydrothermal strategy. In a typical procedure, 15 mL of as-obtained GO suspension (2 mg mL^−1^) was dispersed in 20 mL of DI water and then ultrasonicated for 2 h. Then, 0.393 g of l-cysteine, 0.1139 g of SnCl_4_·5H_2_O and 0.0773 g of CoCl_2_·6H_2_O were added into the above GO suspension under stirring. This mixture was sonicated for 2 h and transferred into a 50-mL Teflon-lined stainless-steel autoclave and kept at 180 °C for 24 h. Then, the obtained product was centrifuged and washed with DI water and absolute ethanol three times, respectively. The final material was dried at 50 °C for 12 h. For comparison, CoS_2_–rGO composite was prepared by the same synthetic route as the SnS_2_@CoS_2_–rGO composite with the same amount of GO, l-cysteine and CoCl_2_·6H_2_O but without the addition of SnCl_4_·5H_2_O. Similarly, SnS_2_–rGO composite was synthesized by the same synthetic route without the addition of CoCl_2_·6H_2_O. Additionally, pure rGO was synthesized without the addition of SnCl_4_·5H_2_O or CoCl_2_·6H_2_O. Composites with different amounts of GO (50 and 20 mg) were also fabricated using the same process as the SnS_2_@CoS_2_–rGO composite. Finally, composites with different ratios of Co to Sn were prepared by adjusting the molar ratio of CoCl_2_·6H_2_O to SnCl_4_·5H_2_O using the same process.

### Characterization and Instruments

The phases of the samples were characterized by powder X-ray diffraction (XRD) (SmartLab, Rigaku) with Cu Kα (*λ* = 1.54178 Å) radiation source. The morphology of the samples was determined by means of scanning electron microscopy (SEM) (JEOL JSM-7800F) and transmission electron microscopy (TEM) (Tecnai G2 F30). The Brunauer–Emmett–Teller (BET) surface area of the sample was measured on a Quantachrome Autosorb-iQ-MP surface area detecting instrument with N_2_ physisorption at 77 K. X-ray photoelectron spectroscopy (XPS) was carried out on a PHI 5600 instrument (PerkinElmer, USA). Raman spectroscopy was recorded on a Renishaw spectrometer. Thermogravimetric analysis (TGA) was performed under air flow by a TG 209 (Netzsch). Atomic force microscope (AFM) measurement was recorded on a XE7, Park system. The atomic ratio of Co to Sn was confirmed by inductively coupled plasma optical emission spectrometry (ICP-OES, Prodigy 7, Leeman Labs). Elemental analysis was tested by a VarioELIII elemental analyser, Elementar, Germany.

### Electrochemical Measurements

At first, the active material, carbon black and carboxymethylcellulose sodium (CMC) binder were fully mixed in a ratio of 70:20:10 (wt%) to form a slurry. Subsequently, the slurry was cast on copper foil by a doctor-blade and dried at 80 °C for 12 h in a vacuum oven. The areal mass loading was around 1.2 mg cm^−2^. The electrochemical tests were carried out in a CR2032 coin cell with Na metal as the counter electrode. The electrolyte solution was 1.0 mol L^−1^ NaClO_4_ in a 1:1 volumetric mixture of propylene carbonate and ethylene carbonate with 5% fluoroethylene carbonate additive. All cells were assembled in an Ar-filled dry box with water and oxygen content lower than 1 ppm. Cyclic voltammetry (CV) curves were obtained by a CHI 660B electrochemical workstation with a scanning rate of 0.2 mV s^−1^ in the voltage range of 0.01 − 3.0 V. The cells were galvanostatically cycled between 0.01 and 3.0 V using various currents at room temperature. Electrochemical impedance spectroscopy (EIS) was carried out after cell cycling in the frequency range of 100 kHz–0.01 Hz with a bias signal amplitude of 0.01 V.

## Results and Discussion

**Fig. 1:**
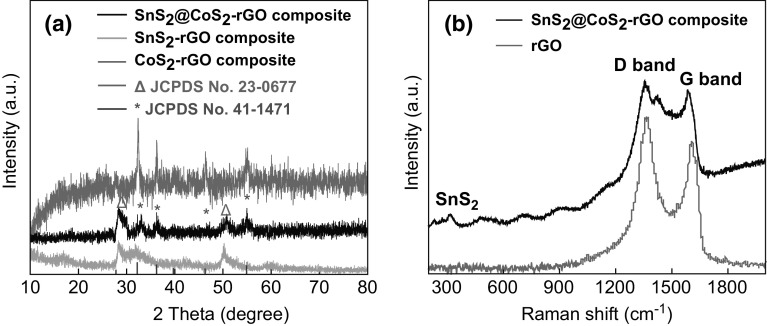
**a** XRD patterns of the SnS_2_@CoS_2_–rGO composite, SnS_2_–rGO composite and CoS_2_–rGO composite and **b** Raman spectra of the SnS_2_@CoS_2_–rGO composite and rGO

The XRD patterns of the as-prepared samples are shown in Fig. 1a. It can be seen that all the diffraction peaks are well indexed to the hexagonal SnS_2_ (JCPDS card No. 23-0677) and cubic CoS_2_ (JCPDS card No. 41-1471) of the SnS_2_–rGO composite and CoS_2_–rGO composite, respectively, in the absence of relevant impurities, revealing pure phases of the SnS_2_ and CoS_2_. For the SnS_2_@CoS_2_–rGO composite, the main reflections are well ascribed to the characteristic peaks of SnS_2_ and CoS_2_. Although the characteristic diffraction peaks of rGO are not observed, this is possibly due to a low degree of crystallinity. Raman spectroscopy further confirms the existence of rGO (Fig. 1b), which presents two peaks centered at 1361 and 1587 cm^−1^, assigned to disordered carbon (D band) and graphitic carbon (G band), respectively [37]. A small peak at 311 cm^−1^ is ascribed to the *A*_1g_ mode of SnS_2_ [28]. Additionally, compared to pure rGO, the D peak of rGO in the SnS_2_@CoS_2_–rGO composite shifted to a slightly lower wavenumber, indicating that the interaction between SnS_2_@CoS_2_ and rGO is strong [38, 39]. The rGO content of the composite was detected by TGA (Fig. S1), which exhibits a weight loss of approximately 0.4% between 25 and 200 °C, attributed to the loss of absorbed water, and a ~ 24.5% decrease from 200 to 900 °C, corresponding to the oxidation of SnS_2_, CoS_2_, and combustion of carbon, in accordance with other metal sulfide/carbon studies [16]. Consequently, the content of carbon in the SnS_2_@CoS_2_–rGO composite was determined to be around 18.6 wt%. In order to further investigate the graphene content, elemental analysis of the SnS_2_@CoS_2_–rGO composite was carried out, which showed the graphene content to be 13.06%. The atomic ratio of Sn and Co was determined to be approximately 1:1 by the ICP-OES method.

**Fig. 2:**
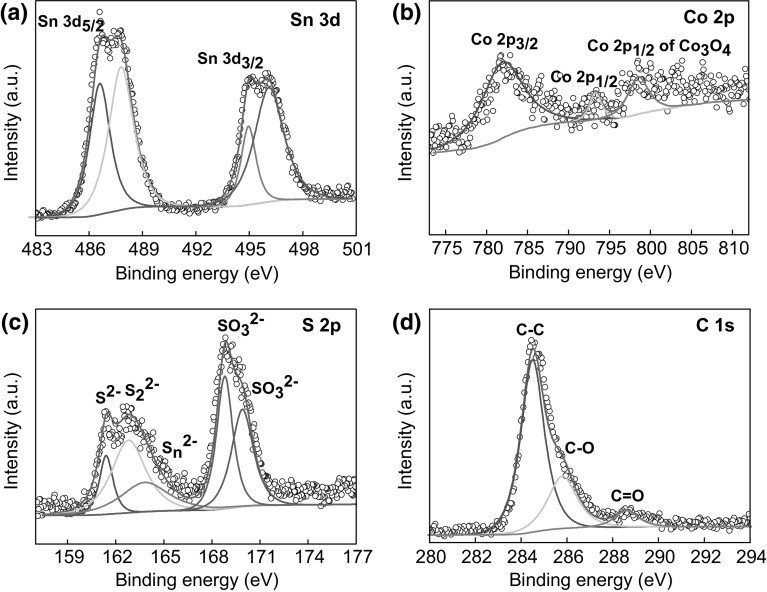
XPS spectra of **a** Sn 3d, **b** Co 2p, **c** S 2p and **d** C 1s core levels of the SnS_2_@CoS_2_–rGO composite

XPS was used to investigate the electronic states and surface composition of the SnS_2_@CoS_2_–rGO composite, presented in Fig. S2 and Fig. 2. Figure S2 shows the survey spectrum, suggesting the existence of Co, Sn, S, and C elements, in accordance with the EDS result. The high-resolution Sn 3d spectrum, shown in Fig. 2a, exhibits two characteristic peaks of SnS_2_ at 487.0 eV (Sn 3d_5/2_) and 495.0 eV (Sn 3d_3/2_) [40], accompanied by two peaks at 486.2 and 496.1 eV, assigned to Sn 3d_5/2_ and Sn 3d_3/2_ of SnS, respectively [41]. The XPS peaks at 781.9 and 793.3 eV in the Co 2p spectrum (Fig. 2b) correspond closely to Co 2p_3/2_ and Co 2p_1/2_ of CoS_2_, respectively, whereas another peak at 798.5 eV is assigned to Co 2p_1/2_ of Co_3_O_4_ [42], mainly resulting from partial surface oxidation of the composite [43]. The S 2p spectrum (Fig. 2c) exhibits five peaks at around 161.4, 162.8, 163.8, 168.8, and 169.9 eV. The first three peaks are attributed to characteristic peaks of S^2−^, S_2_^2−^, and S_n_^2−^, respectively, indicating the presence of S^−^ and S^2−^. Meanwhile, the other two peaks at 168.8 and 169.9 eV correspond to S 2p_3/2_ and S 2p_1/2_ of SO_3_^2−^, respectively [16]. Three peaks in the C 1s spectrum at 284.6, 285.9, and 288.7 eV are observed in Fig. 2d, which are attributed to *sp*^2^ C–C, C-O, and O-C = O bonds, respectively [44].

**Fig. 3:**
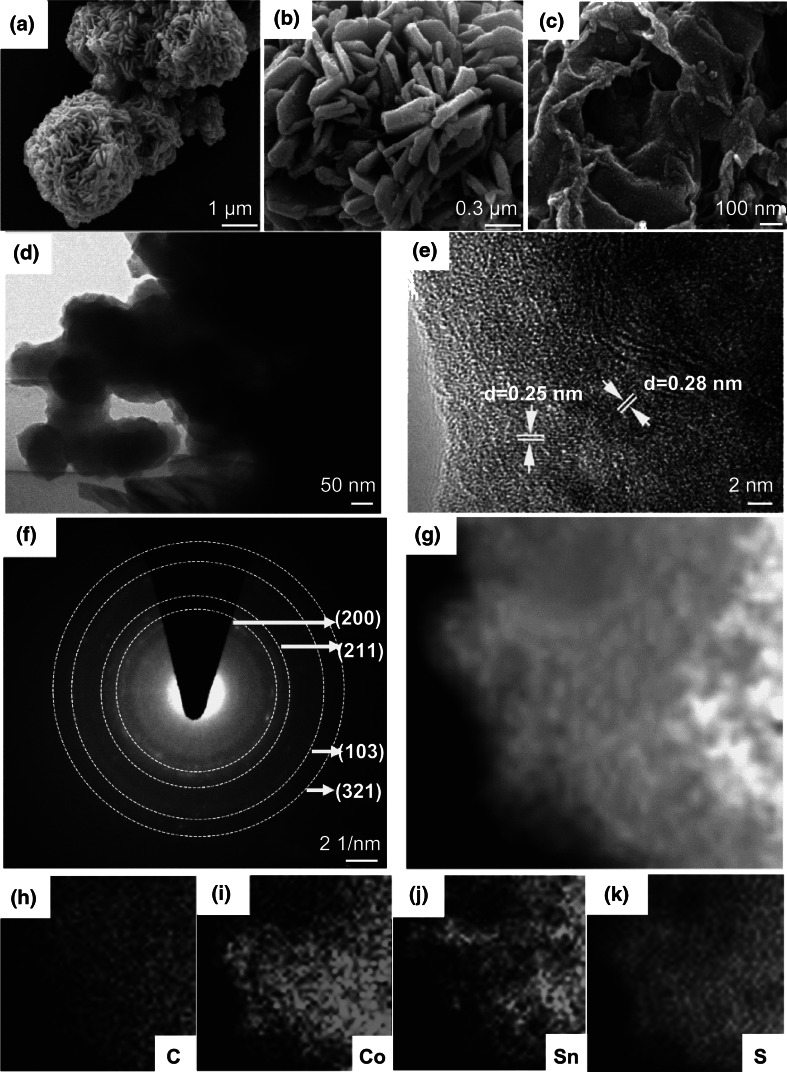
**a**–**c** low- and high-magnification SEM images, **d** TEM image, **e** HRTEM image, **f** SAED pattern and **g**–**k** STEM image and corresponding element mappings of the SnS_2_@CoS_2_–rGO composite

The morphology and microstructure of the SnS_2_@CoS_2_–rGO composite was investigated using SEM, TEM, HRTEM, and STEM, as shown in Fig. 3. It can be seen that the SnS_2_@CoS_2_–rGO composite displays flower-like spherical morphology, composed of nanosheets with random orientation (Fig. 3a, b). In the high-magnification micrograph (Fig. 3c), a large number of nanoparticles with an average size of approximately 45 nm are decorated on the nanosheets. For the SnS_2_–rGO composite, hierarchical spheres consisting of nanosheets are formed (Fig. S3a). There are no individual SnS_2_ particles; SnS_2_ sheets are well dispersed on the rGO (Fig. S3b). Compared with the SnS_2_–rGO composite, the CoS_2_–rGO composite exhibits rough rGO sheets (Fig. S3c) over which CoS_2_ nanoparticles with a mean size of around 50 nm are dispersed (Fig. S3d), in agreement with other reports [45]. Therefore, the SnS_2_@CoS_2_–rGO composite appears to be composed of SnS_2_ nanosheets and CoS_2_ nanoparticles, in combination with rGO nanosheets.

Furthermore, the TEM image (Fig. 3d) of the SnS_2_@CoS_2_–rGO composite indicates CoS_2_ nanoparticles with a size of approximately 50 nm on the SnS_2_ nanosheets. The HRTEM image (Fig. 3e) displays two lattice fringes of 0.23 and 0.28 nm attributed to the (101) and (211) planes of SnS_2_ and CoS_2_, respectively, accompanied by a disordered lattice fringe of rGO. Meanwhile, the corresponding diffraction rings (Fig. 3f) indicate the polycrystalline nature of the SnS_2_@CoS_2_–rGO composite. Additionally, the elemental mapping of the SnS_2_@CoS_2_–rGO composite shows that Co, Sn, S, and C are uniformly distributed in the composite, suggesting homogeneous decoration of the SnS_2_@CoS_2_–rGO composite (Fig. 3g–k).

**Fig. 4:**
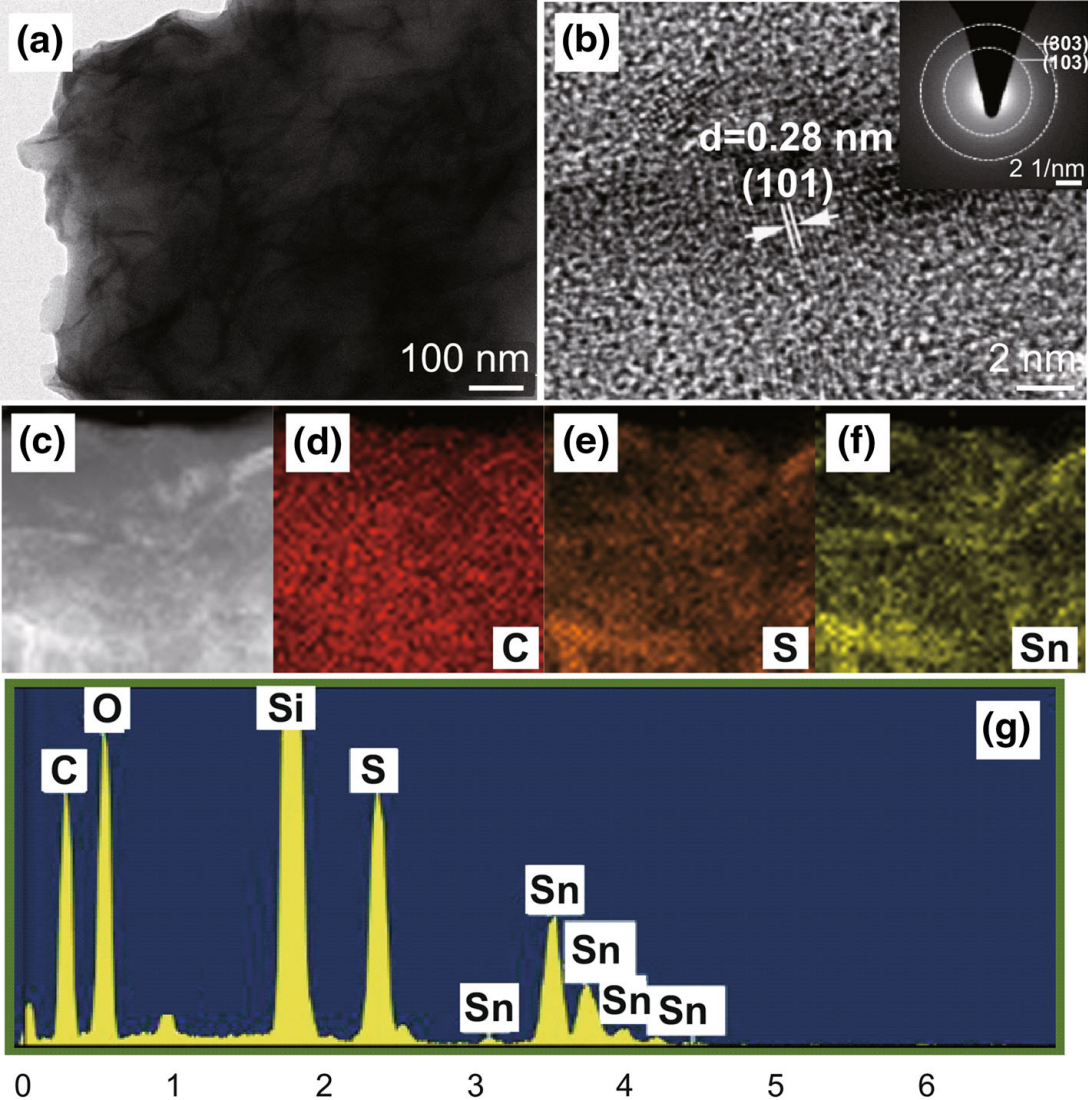
**a** TEM image, **b** HRTEM image (inset is SAED pattern), **c**–**f** STEM image and corresponding element mappings of the SnS_2_–rGO composite and **g** EDS spectrum of SnS_2_–rGO composite

**Fig. 5:**
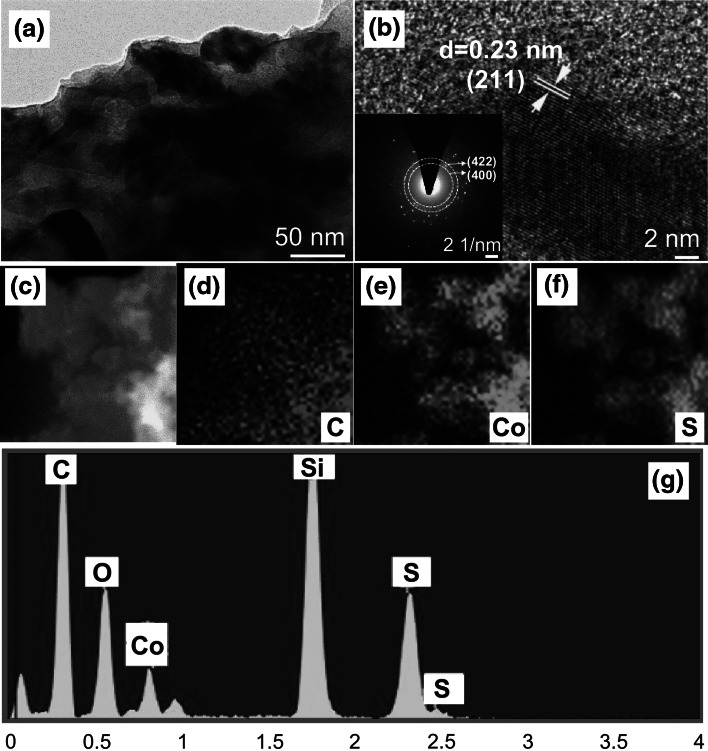
**a** TEM image, **b** HRTEM image (inset is SAED pattern), **c**–**f** STEM image and corresponding element mappings of the CoS_2_–rGO composite and **g** EDS spectrum of CoS_2_–rGO composite

Figure 4 shows TEM, HRTEM, SAED, mapping images, and EDS spectrum of the SnS_2_–rGO composite. The SnS_2_ nanosheets are dispersed over the rGO (Fig. 4a) and the corresponding HRTEM image reveals a lattice spacing of 0.28 nm, corresponding to the (101) plane of SnS_2_ (Fig. 4b). The SAED image (inset of Fig. 4b) indicates the polycrystalline nature of the SnS_2_–rGO composite, in which the elements of Sn, S, and C are uniformly dispersed, as seen from the mapping images (Fig. 4c–f). In addition, the ratio of Sn:S was calculated to be 1:2.02 from Fig. 4g. Additionally, CoS_2_ nanoparticles with a diameter size of around 55 nm are decorated on the rGO, observed from the TEM image of the CoS_2_–rGO composite (Fig. 5a), in agreement with the SEM image. The HRTEM image exhibits a lattice fringe of 0.23 nm, indexed to the (211) plane of CoS_2_ (Fig. 5b) and the corresponding SAED also presents the polycrystalline nature of the CoS_2_–rGO composite (inset of Fig. 5b). The Co, S, and C elements are uniformly distributed in the CoS_2_–rGO composite, as demonstrated by the elemental mappings (Fig. 5c–f), and the ratio of Co:S was calculated to be 1:2.01, which further demonstrates that the obtained product is CoS_2_ (Fig. 5g). Meanwhile, the thickness of the rGO nanosheets in the three composites was also determined from the AFM image shown in Fig. S4. It can be clearly seen that the thickness of the rGO sheet is ~ 4 nm and hence each sheet may be nine or ten layers.

To further investigate the porous nature of the SnS_2_@CoS_2_–rGO composite, nitrogen adsorption/desorption measurement was performed. Typical IV nitrogen isotherms combined with a large hysteresis loop in the pressure range of 0.5–1.0 (P/P_0_) (Fig. S5a) reveal the existence of mesopores with a mean size of approximately 5.8 nm (Fig. S5b). Furthermore, the BET specific surface area was calculated to be 12.6 m^2^ g^−1^, which would be beneficial for the improvement of electrochemical performance resulting from good interfacial contact between electrolytes and electrode. Consequently, a high sodium-storage ability would be expected [36].

The sodium-storage performance of the SnS_2_@CoS_2_–rGO composite electrode was evaluated to confirm its great promise for SIBs in comparison with those of the SnS_2_–rGO and CoS_2_–rGO composite electrodes. The electrochemical process of the SnS_2_–rGO composite electrode was studied by cyclic voltammograms (CV) in the voltage range of 0.01–3 V at a scan rate of 0.2 mV s^−1^ and is presented in Fig. S6a. For the first cycle, two reduction peaks are clearly seen at around 1.59 and 1.29 V, which originate from the intercalation of sodium ions into the SnS_2_ layers without phase decomposition [46]. Moreover, a broad reduction peak ranging from 0.2 to 0.6 V is attributed to a conversion reaction to form metallic Sn and Na_2_S, an alloying reaction to produce Na_*x*_Sn [47], and the formation of a solid electrolyte film (SEI) [48]. In subsequent cycles, this peak gradually shifted to 0.35 V. In addition, the reduction peak at 0.01 V corresponds to Na^+^ storage on the rGO. For the first anodic scan, the oxidation peak at 1.25 V corresponds to the dealloying reaction of Na_*x*_Sn. Further, this peak overlapped well in all cycles, which suggests that the alloying/dealloying reaction is highly reversible. Meanwhile, the anodic peak at 2.1 V can be attributed to the oxidation of Sn to form SnS_2_ and the extrusion of Na^+^ from Na_*x*_SnS_2_ without phase decomposition [49]. Furthermore, the anodic peak at 1.64 V results from Na^+^ release on the rGO [28].

**Fig. 6:**
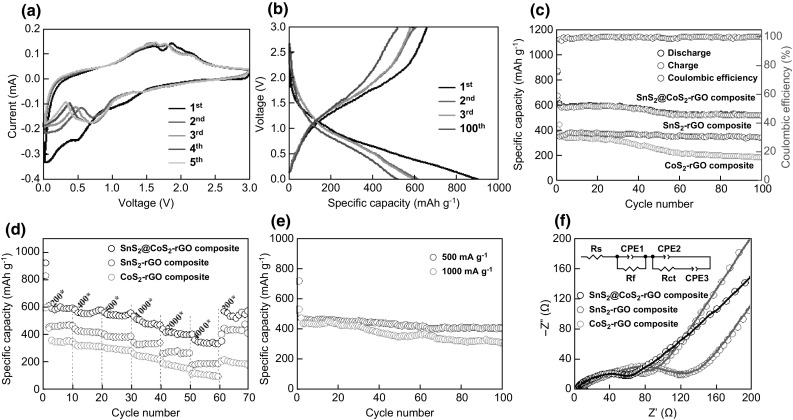
**a** CV curves of the SnS_2_@CoS_2_–rGO composite, **b** charge/discharge profiles for the initial three cycles and the 100th cycle of the SnS_2_@CoS_2_–rGO composite at a current density of 200 mA g^−1^, **c** cycling performance of the SnS_2_@CoS_2_–rGO composite, SnS_2_–rGO composite, CoS_2_–rGO composite at a current density of 200 mA g^−1^, and the corresponding coulombic efficiency of the SnS_2_@CoS_2_–rGO composite, **d** Rate capability of the SnS_2_@CoS_2_–rGO, SnS_2_–rGO and CoS_2_–rGO composite electrodes at varied current densities from 100 to 4000 mA g^−1^, **e** capacity versus cycle number of the SnS_2_@CoS_2_–rGO composite at higher current densities of 500 and 1000 mA g^−1^ and **f** Nyquist plots and fitted results of the SnS_2_@CoS_2_–rGO composite, SnS_2_–rGO composite, and CoS_2_–rGO composite after the 100th cycle at discharged state. Inset: equivalent circuit model of the studied system

For the CoS_2_–rGO composite electrode (Fig. S6b), in the first cycle, three significant reduction peaks centered at 0.86, 0.57, and 0.43 V can be ascribed to the insertion of Na, the conversion reaction to the formation of Co and Na_2_S, and the formation of SEI film, respectively [15]. In the subsequent scan, the first two reduction peaks shifted to 1.44 and 0.84 V, respectively, originating from the enhanced kinetics of the electrode. Moreover, two oxidation peaks at 1.83 and 2.04 V may originate from the oxidation of Co and the de-insertion of Na to form CoS_2_, respectively [29]. Figure 6a shows the CV curves for the SnS_2_@CoS_2_–rGO composite. A broad reduction peak ranging from 1.40 to 0.95 V is observed, which may be related to the intercalation of sodium ions into the SnS_2_ and CoS_2_ without phase decomposition for the first cycle, due to the nature of the SnS_2_@CoS_2_–rGO composite. Another evident reduction peak located at around 0.50 V may arise from the conversion reaction to form metallic Sn and Na_2_S, the alloying reaction to produce Na_*x*_Sn, and the formation of metallic Co. In addition, a reduction peak at 0.07 V corresponds to sodium-ion storage on the rGO. A weak oxidation peak at 1.25 V is observed, which is ascribed to the dealloying reaction of Na_*x*_Sn to form Sn. In addition, an obvious oxidation peak at 1.86 results from the oxidation of metallic Co [15]. Another oxidation peak at 2.16 V is ascribed to oxidation of Sn and exclusion of Na^+^ from the Na_*x*_SnS_2_, associated with an oxidation peak at 1.63 V related to the release of Na^+^ from rGO. In the subsequent cycles, the reduction peaks at 0.50 and 1.40–0.99 V shift gradually to 0.70 and 1.46 V, and the corresponding oxidation peaks also move, which is different from the first cycle, in accordance with previously reported results [15, 50]. Further, the CV curves from the second cycle to the fifth cycle overlap well, suggesting excellent cycling stability and reversibility of the SnS_2_@CoS_2_–rGO composite in the sodium-storage process.

Figure 6b presents the galvanostatic charge/discharge voltage profiles of the SnS_2_@CoS_2_–rGO composite electrode at 200 mA g^−1^, which is consistent with the CV results. The first discharge capacity was around 895.3 mAh g^−1^ below 1.5 V, and the corresponding first charge capacity was approximately 652.8 mAh g^−1^ with two weak voltage plateaus at 0.9–1.3 and 1.6–1.8 V. Therefore, the initial coulombic efficiency was approximately 72.9%. An initial irreversible capacity loss mainly arises from the presence of SEI film [51], structural re-arrangement of the electrode in the conversion process [52], and electrolyte intercalation into the rGO [53]. Additionally, the second discharge and charge delivered capacities were 611.6 and 600.2 mAh g^−1^, respectively, while the third discharge and charge capacities were 595.4 and 578.2 mAh g^−1^, respectively. After 100 cycles, the charge capacity remained at 514.0 mAh g^−1^ with a coulombic efficiency of 99.5%. The trend of the discharge and charge capacities in combination with corresponding efficiency over 100 cycles at a current density of 200 mA g^−1^ is presented in Fig. 6c. As can be seen, the SnS_2_@CoS_2_–rGO composite electrode possesses a steady capacity of up to 514.0 mAh g^−1^ after 100 cycles. Additionally, the initial coulombic efficiencies of the SnS_2_–rGO composite and CoS_2_–rGO composite are 47.5 and 62.2%, respectively (Fig. S6c). In comparison with the low reversible capacities of the SnS_2_–rGO composite (340.5 mAh g^−1^) and CoS_2_–rGO composite (180.5 mAh g^−1^) (Fig. 6c), the good sodium-storage performance of the SnS_2_@CoS_2_–rGO composite shows that the combination of CoS_2_ and SnS_2_ plays a synergistic effect in creating a high-performance material for SIBs. In contrast, pure rGO without SnS_2_ and CoS_2_ tested under the same conditions presents a low capacity of 220 mAh g^−1^ after 100 cycles (Fig. S6d), thus revealing the prominent contribution of SnS_2_ and CoS_2_ in the composite electrode. Additionally, compared with previously reported tin sulfide and cobalt sulfide-based anodes, the present material manifests great competitiveness in SIB applications (Table 1).

**Table 1:** Comparison of the Na-storage performance of reported tin sulfide and cobalt sulfide-based materials

Materials	First capacity loss (mAh g^−1^)	Cycle number	Current density (mA g^−1^)	Mass loading (mg cm^−2^)	Charge capacity (mAh g^−1^)	References
SnS_2_@CoS_2_–rGO composite	242.5	100	200	1.2	514.0	This work
	277.6	100	500	1.2	405.8	
SnS_2_-NGS	313.9	100	200	1.6 ± 0.2	450	[25]
SnS_2_/C	440	100	50		600	[23]
Co_3_S_4_@PNAI nanotubes	~178.8	100	200	1.75	252.5	[17]
NiS_2_@CoS_2_@C@C nanocubes		250	1000		600	[34]
CoS_2_/rGO composite	261	100	100	1–1.2	400	[54]
CoS_2_/carbon composite	623.5	120	100		610	[55]

The SnS_2_@CoS_2_–rGO composite also displayed excellent rate capability, as shown in Figs. S6d, S7a. As the current density increases from 200 to 4000 mA g^−1^, the discharge capacity slightly drops. When the current density reached 200, 400, 500, 1000, 2000, and 4000 mA g^−1^, the specific capacities were 588, 576, 552, 468, 396, and 330 mAh g^−1^, respectively, all of which were higher than those of SnS_2_–rGO and CoS_2_–rGO composites. Even after testing at 4000 mA g^−1^, a high reversible capacity similar to the initial value could be achieved when returning the current density to 200 mA g^−1^. At higher current densities of 500 and 1000 mA g^−1^, the SnS_2_@CoS_2_–rGO composite possesses reversible capacities of 405.8 and 309.8 mAh g^−1^ with only minor decay over 100 cycles, respectively (Fig. 6e), indicating high rate capability and good cycling stability for the SnS_2_@CoS_2_–rGO composite. The improved Na-storage performance of the SnS_2_@CoS_2_–rGO composite electrode can be attributed to two factors. First, rGO not only improves electronic conductivity of the composite but also accommodates the large volume variation during the charge/discharge process. Secondly, the synergetic effect of SnS_2_ and CoS_2_ can offer an optimal electrode/electrolyte interface [42].

In order to further reveal the transport kinetics for the SnS_2_@CoS_2_–rGO composite, EIS spectra were carried out and the corresponding data are shown in Fig. 6f after 100 cycles at discharged state. The impedance spectra consist of a depressed semicircle in the high-frequency region corresponding to the SEI resistance (*R*_f_) and charge transfer impedance (*R*_ct_) within the electrode/electrolyte interface, accompanied by a sloping line in the low frequency region associated with diffusion of Na^+^ within the electrode [56]. By fitting the Nyquist plot of the impedance spectroscopy using an equivalent circuit (inset of Fig. 6f), the value of *R*_f_ + *R*_ct_ for the SnS_2_@CoS_2_–rGO composite (54.90 Ω) was lower than those of the SnS_2_–rGO composite (89.39 Ω) and CoS_2_–rGO composite (121.11 Ω) (Fig. 6f), suggesting that electron transfer in the SnS_2_@CoS_2_–rGO composite electrode is easier. Moreover, EIS spectra of the SnS_2_@CoS_2_–rGO composite after various cycles at a current density of 1000 mA g^−1^ are shown in Fig. S7b. It can be seen that the semicircles in the high-frequency range present no obvious change after the 1st, 10th, 30th, or 100th cycles, indicating that there is hardly any significant change of the charge transfer resistance of Na ions to and from the electrodes. Therefore, the cycling performance of the SnS_2_@CoS_2_–rGO composite electrode is good [57–59].

The influence of the amount of rGO on the sodium-storage performance of the SnS_2_@CoS_2_–rGO composite was also studied. As shown in Fig. S8a, the capacity of the composite with 20 mg rGO gradually drops upon cycling and stays at 345.8 mAh g^−1^ after 100 cycles, which derives from insufficient rGO to buffer the volume change. Additionally, when increasing the amount of rGO to 50 mg, the SnS_2_@CoS_2_–rGO composite possesses stable cycling performance, but low specific capacity (only 419.7 mAh g^−1^ after 100 cycles). The excessive rGO could act as a good buffer layer; however, its theoretical sodium-storage capacity is low. As a result, the optimal rGO content is of vital importance for the SnS_2_@CoS_2_–rGO composite electrode; when the amount of rGO is 30 mg, the sodium-storage performance should be best.

Samples with different ratios of Co to Sn were also prepared by adjusting the molar ratio of CoCl_2_·6H_2_O:SnCl_4_·5H_2_O, and the corresponding sodium-storage performance was shown in Fig. S8b. The capacity of the composite with atomic ratios of Co: Sn of 1:4 and 1:2 gradually drop upon cycling and stay at 289.2 and 352.2 mAh g^−1^ after 100 cycles. When this ratio is changed to 2:1, 4:1, or 8:1, the SnS_2_@CoS_2_–rGO composite possesses stable cycling performance, but somewhat low specific capacity (only 489.4, 454.6, and 435.3 mAh g^−1^ after 100 cycles, respectively). As a result, when the atomic ratio of Co:Sn was 1:1, the sodium-storage performance was best.

The morphology and structure of the SnS_2_@CoS_2_–rGO composite after the charge/discharge process are shown in Fig. S9. Figure S9a, b indicates that the nanoparticles are decorated on the rGO. There are two lattice fringes of 0.313 and 0.214 nm, attributed to the (100) and (102) planes of SnS_2_ and a lattice spacing of 0.277 nm, ascribed to the (200) plane of CoS_2_ (Fig. S9c). Lattice fringes of SnS_2_ and CoS_2_ are also observed in Fig. S9d, which indicates that the SnS_2_ and CoS_2_ can be regenerated after 100 cycles, which is the key factor for cycling stability of the SnS_2_@CoS_2_–rGO composite [60].

## Conclusions

In summary, a SnS_2_@CoS_2_–rGO composite was synthesized by a facile hydrothermal strategy. The obtained composite, assembled with SnS_2_ nanosheets and CoS_2_ nanoparticles along with rGO sheets with good contact interface, possessed high specific capacity, excellent rate capability, and good cycling stability (514.0 mAh g^−1^ at 200 mA g^−1^ after 100 cycles) as an anode material for SIBs. The excellent performance of the SnS_2_@CoS_2_–rGO composite can be ascribed to the rGO acting as a highly conductive matrix and the combined effect of SnS_2_ and CoS_2_, which are beneficial for mitigating the volume change and offer fast electron transport due to good electronic contact. Consequently, the SnS_2_@CoS_2_–rGO composite is a promising candidate for application in SIBs.

## Electronic supplementary material

Below is the link to the electronic supplementary material.

Supplementary material 1 (PDF 858 kb)
