# Supplementary material for: Improved Electrochemical Performance Based on Nanostructured SnS2@CoS2–rGO Composite Anode for Sodium-Ion Batteries
Source: Nanomicro Lett. 2018 Apr 13;10(3):46. doi: 10.1007/s40820-018-0200-x (PMC6199098; doi:10.1007/s40820-018-0200-x)
Supplement: Supplementary file 1 — Supplementary material 1 (PDF 858 kb) [file 40820_2018_200_MOESM1_ESM.pdf]

Supporting Information for

Improved Electrochemical Performance Based on Nanostructured SnS_2@CoS_2-rGO Composite Anode for Sodium-Ion Batteries

Xia Wang[1,*], Xueying Li[1], Qiang Li[1], Hongsen Li[1], Jie Xu[1], Hong Wang[1], Guoxia Zhao[1], Lisha Lu[1], Xiaoyu Lin[1], Hongliang Li[2], Shandong Li[1,*]

[1]College of Physics, Key Laboratory of Photonics Materials and Technology in Universities of Shandong, and Laboratory of Fiber Materials and Modern Textile, the Growing Base for State Key Laboratory, Qingdao University, Qingdao 266071, People's Republic of China

[2]Institute of Materials for Energy and Environment, Qingdao University, Qingdao 266071, People's Republic of China

*Corresponding authors. E-mail: lishd@qdu.edu.cn (Shandong Li), wangxiakuaile@qdu.edu.cn (Xia Wang)

Supplementary Figures

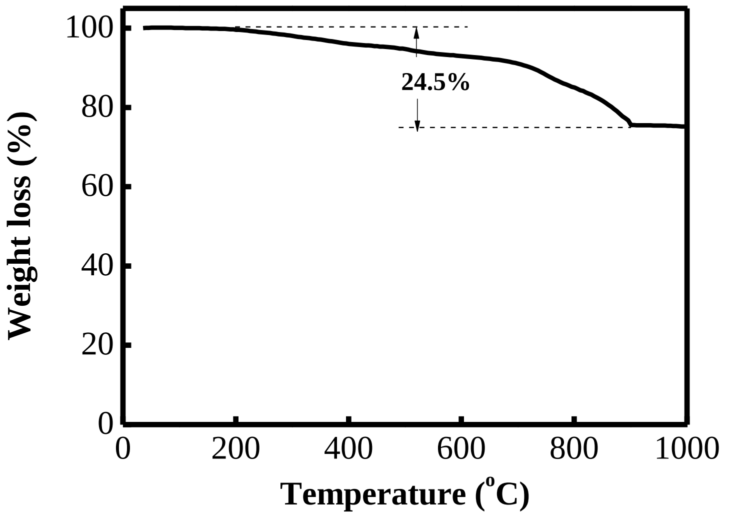

Fig. S1 Thermogravimetric analysis profile of the SnS_2@CoS_2-rGO composite

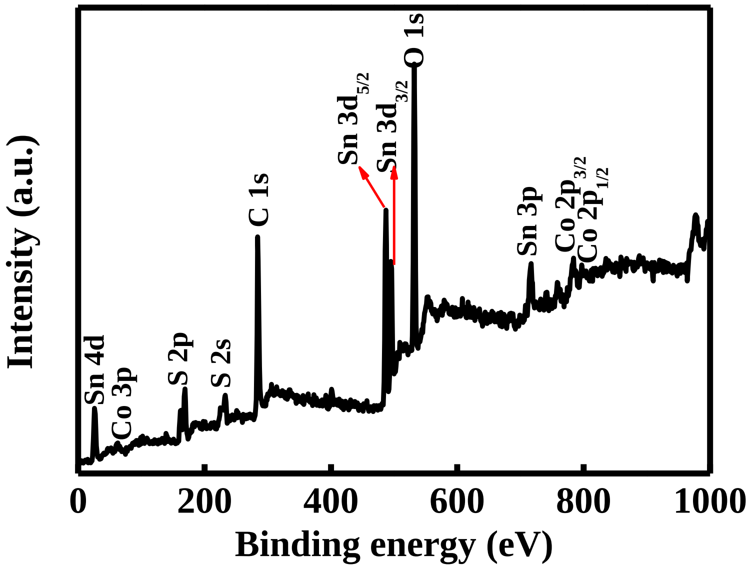

Fig. S2 Survey XPS spectra of the SnS_2@CoS_2-rGO composite

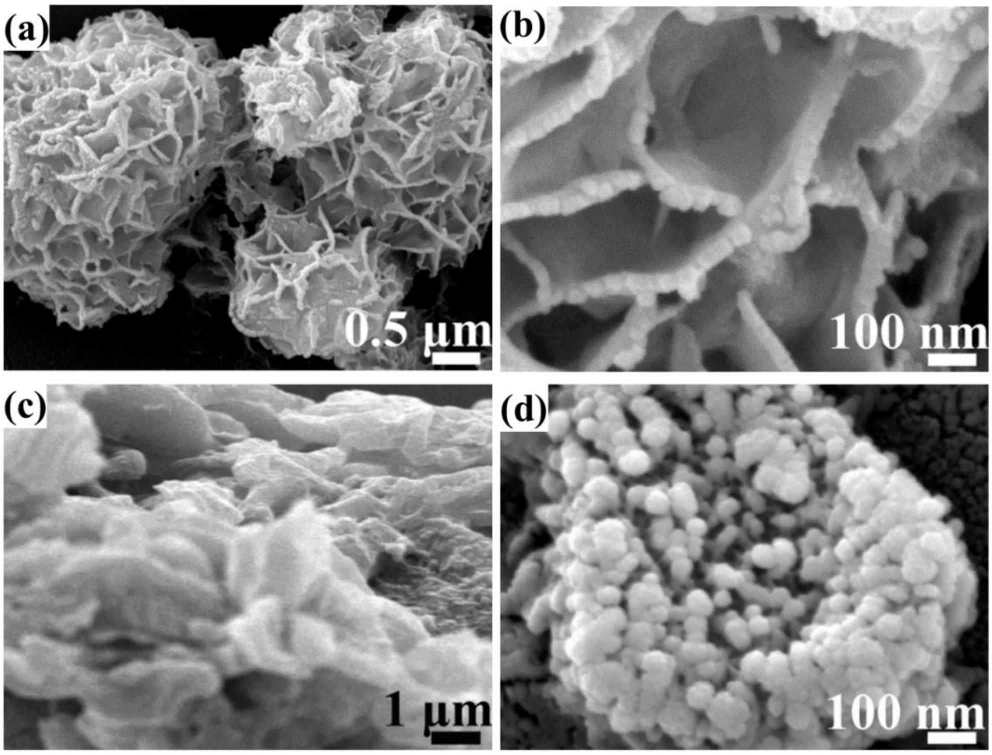

Fig. S3 Low- and high- magnification SEM images of **a, b** SnS_2-rGO composite and **c, d** CoS_2-rGO composite

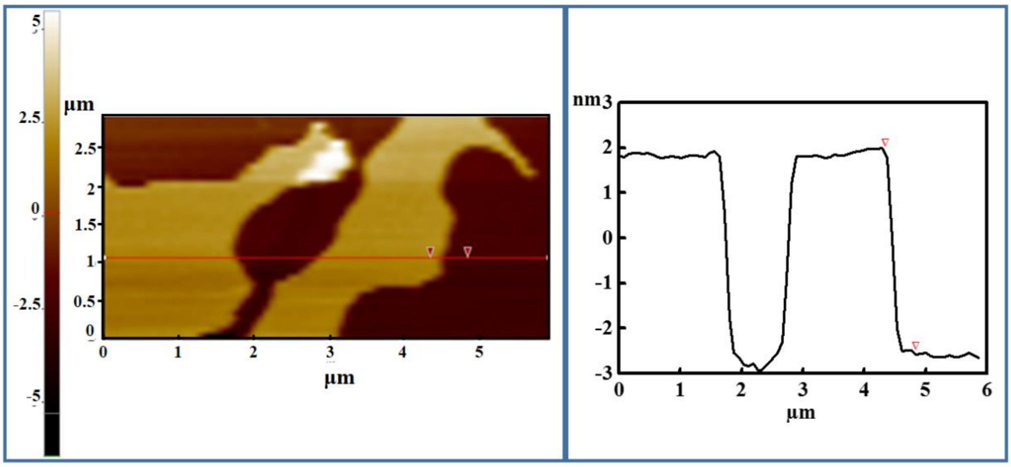

Fig. S4 AFM image and corresponding height image of the rGO

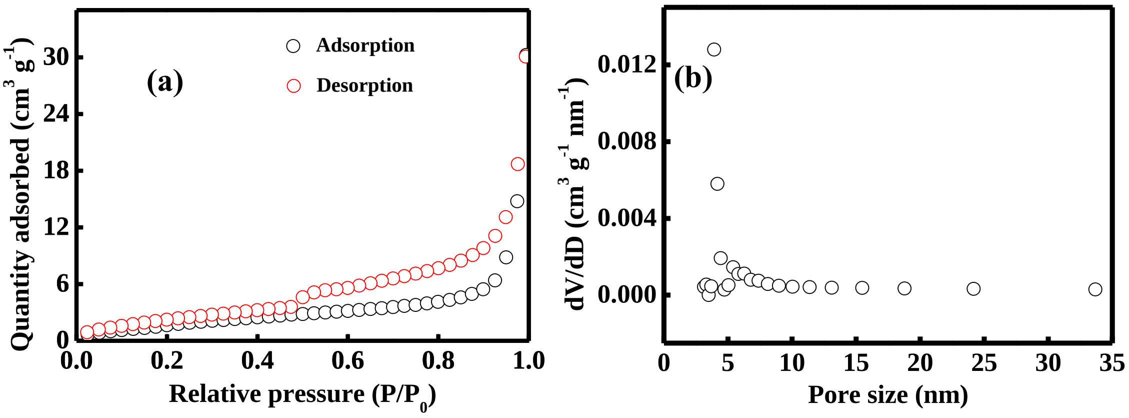

Fig. S5 a N_2 adsorption–desorption isotherms and **b** pore size distribution curves of the SnS_2@CoS_2-rGO composite

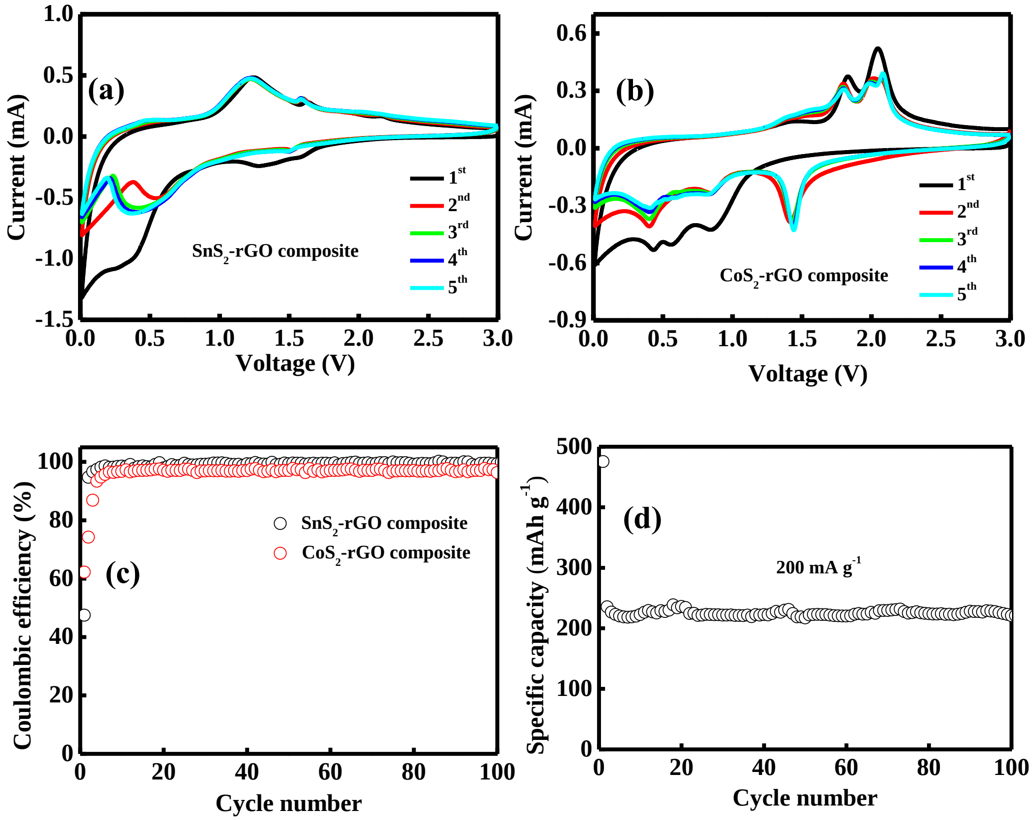

Fig. S6 a, b CV curves of the SnS_2-rGO composite and CoS_2-rGO composite, respectively. **c** Initial Coulombic efficiency of the SnS_2-rGO and CoS_2-rGO composites at 200 mA g^{-1}. **d** Cycling performance of the rGO at a current density of 200 mA g^{-1}

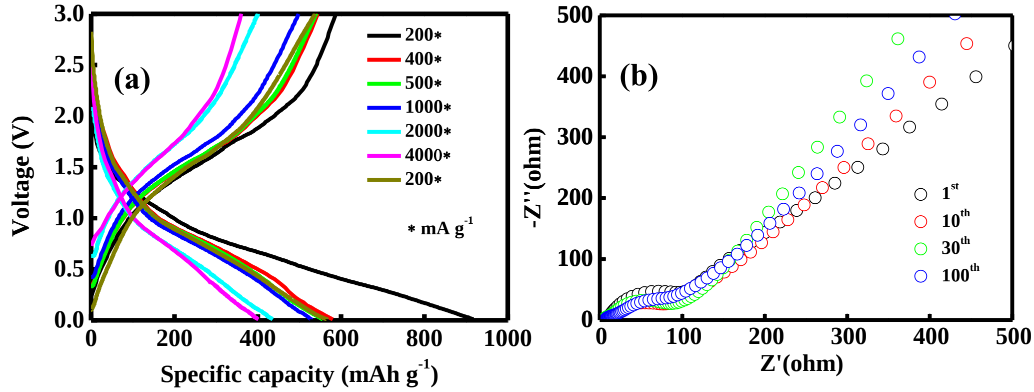

Fig. S7 a Charge/discharge profiles of the SnS_2@CoS_2-rGO composite at various current densities. **b** Nyquist plots of the SnS_2@CoS_2-rGO composite after 1st, 10th, 30th, and 100th cycles at a current density of 1000 mA g^{-1}

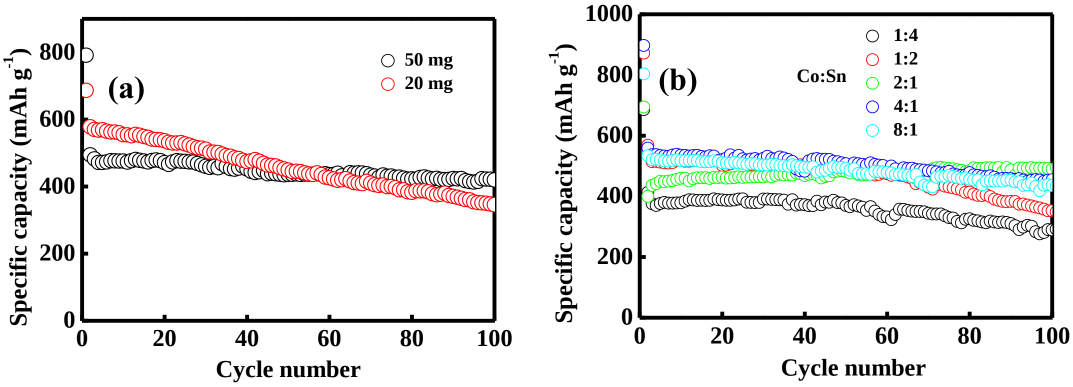

Fig. S8 Cycling performance of the SnS_2@CoS_2-rGO composite with different molar ratio of the $CoCl_2 \cdot 6H_2O$ to $SnCl_4 \cdot 5H_2O$ (**a**) and different amounts of rGO (**b**) at a current density of 200 mA g^{-1}

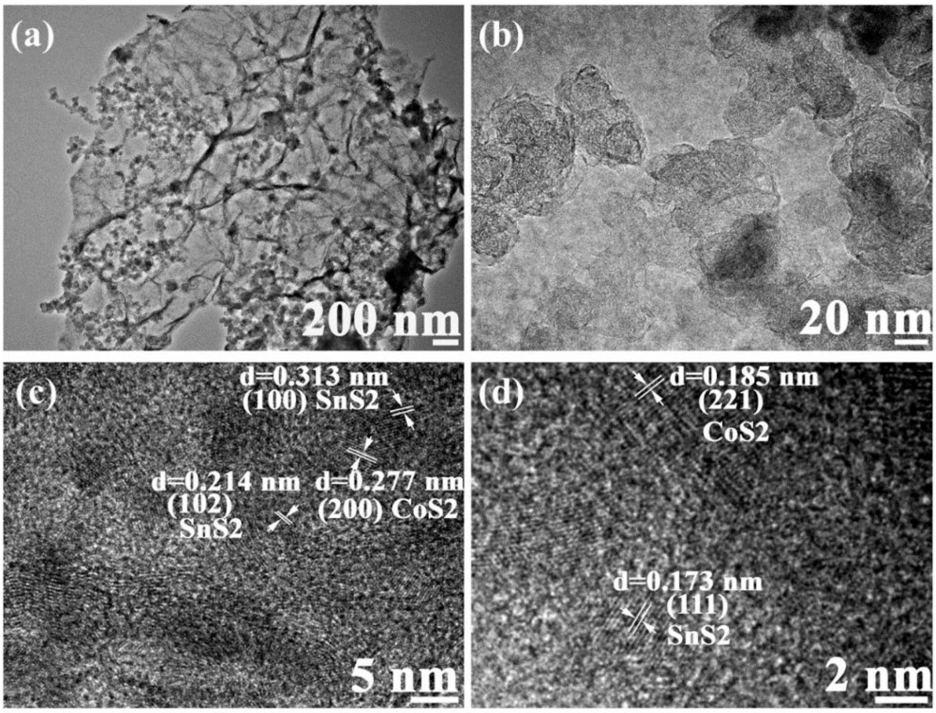

Fig. S9 TEM images of the SnS_2@CoS_2-rGO composite after 100 cycles at a current density of 200 mA g^{-1}
